# Strategies targeting PD-L1 expression and associated opportunities for cancer combination therapy

**DOI:** 10.7150/thno.80091

**Published:** 2023-03-05

**Authors:** Shuangneng Yin, Zhaojun Chen, Dugang Chen, Dan Yan

**Affiliations:** 1 Institute of Pharmaceutical Innovation, School of Medicine, Hubei Province Key Laboratory of Occupational Hazard Identification and Control, Wuhan University of Science and Technology, Wuhan, Hubei, China.; 2 State Key Laboratory of Biogeology and Environmental Geology, Faculty of Materials Science and Chemistry, China University of Geosciences, Wuhan 430074, China.; 3 Key Laboratory for Green Chemical Process of Ministry of Education, School of Chemical Engineering and Pharmacy, Wuhan Institute of Technology, Wuhan 430205, China.; 4 Wuhan Asia Heart Hospital, Wuhan University of Science and Technology, Wuhan, Hubei, China.; 5 Department of Pathology, Medical College, Wuhan University of Science and Technology, Wuhan, Hubei, China.

**Keywords:** PD-L1, immune checkpoint blockade, targeted therapy, immunotherapy, combination therapy

## Abstract

Immunotherapy has achieved great success recently and opened a new avenue for anti-tumor treatment. Programmed cell death 1/programmed cell death ligand 1 (PD-1/PD-L1) are typical immune checkpoints that transmit coinhibitory signals, muting the host immunity. Monoclonal antibodies that block PD-1/PD-L1 axis have benefited many patients with different tumor diseases. However, the objective response rate is still unsatisfactory. In this review, we summarize three strategies targeting PD-L1 based on different forms of PD-L1 and various regulating mechanisms to enhance the therapeutic effect, including blockade of the interaction between PD-L1 and PD-1, downregulation of PD-L1 expression and degradation of mature PD-L1. Thereinto, we describe a variety of materials have been designed to target PD-L1, including antibodies, nanoparticle, peptide, aptamer, RNA, and small molecule. Additionally, we list the drugs with PD-L1 regulation capacity used in clinical and ongoing studies to explore other alternatives for targeting PD-L1 besides anti-PD-L1 monoclonal antibodies. Moreover, we discuss associated opportunities for cancer combination therapy with other modalities such as chemotherapy, radiotherapy, photodynamic therapy (PDT) and photothermal therapy (PTT), as these conventional or emerging modalities are capable of increasing the immune response of tumor cells by altering the tumor microenvironment (TME), and would display synergistic effect. At last, we give a brief summary and outlook regarding the research status and future prospect of immunotherapy.

## Introduction

Cancer is one of the major threats to human health, and has caused a total death of around 10 million worldwide in 2020, accompanied by another 20 million new cases 1. Currently, various tumors are extensively treated by surgery, radiotherapy and chemotherapy with established standards of care, but the radical cure of tumors with theses modalities are still far from satisfactory due to their individual limitations. The emergence and prevalence of immunotherapy with prominent specificity and low toxicity provides new promise for tumor patients, and is considered as the fourth pillar of tumor treatment.

Immunotherapy capitalizes on the patient's own immune system to fight against tumors, capable of tissue-specific and durable response. As a research hotspot, different forms of immunotherapy are being studied with some of them already being clinically translated, including adoptive cell therapy 2, immune checkpoint blockade (ICB) 3, 4, tumor vaccine 5 and so on. Although immune cells should recognize and eradicate the “foreign” cancer cells, cancer cells can escape the immune surveillance through multiple mechanisms, among which immune checkpoint pathway plays a pivotal role 6. Cytotoxic T lymphocyte-associated antigen 4 (CTLA-4) will translocate to the T cell surface from intracellular environments upon T cell receptor engagement, acting as a coinhibitory factor to hinder the proliferation and activation of T cells. The blocking of CTLA-4 checkpoint could restore the T cell priming and activation to attack cancer cells 7. Based on this mechanism, the first anti-immune checkpoint drug, Ipilimumab, was approved by US Food and Drug Administration (FDA) in 2011 demonstrating significant efficacy in extending the survival of patients with metastatic melanoma. While this mechanism of action normally works in the early stage of the immune response, displaying relatively low response rate and obvious toxicity. Another important immune checkpoint is the PD-1 that is expressed on the antigen-specific T cells as a coinhibitory receptor. The combination of PD-1 with its ligand, the programmed cell death protein ligand 1 (PD-L1), which expresses on cancer cells in the tumor microenvironment (TME), will pass on regulatory signals to effector T cells causing T cell exhaustion and antiapoptotic signals to tumor cells resulting in tumor survival, hence severely suppressing the immune response. So far, More than 10 antibodies have been granted for therapy in multiple tumors 8, and over 1000 clinical trials are being implemented targeting the PD-1/PD-L1 axis 9, for example, in melanoma 10, breast cancer 11, 12, genitourinary carcinoma 13, 14, head and neck cancer 15, hepatocellular carcinoma 16, non-small cell lung cancer (NSCLC) 17, 18, etc., clearly showing the considerable value of anti-PD-1/anti-PD-L1 therapy. However, only a small fraction of people could benefit from the therapy, partially due to the primary or acquired resistance of tumor cells. Compared to the expression of PD-1 mainly limited to certain cell types (e.g. activated T cells, dendritic cells, monocytes), PD-L1 is constitutively expressed by a diverse range of haematopoietic cells, including macrophages, dendritic cells (DCs), T cells, B cells and mast cells, and some non-haematopoietic cell types such as vascular endothelial cells 6. Additionally, extensive expression of PD-L1 on tumor cells and other cells in the TME is of major clinical relevance, therefore more flexible strategies might be allowed to modulate the immune response through targeting PD-L1.

Emerging clinical data and experimental studies have highlighted the PD-L1 expression in tumor is upregulated in response to various anti-tumor therapies, such as chemotherapy 19, radiotherapy 20, PDT 21, PTT 22, SDT 23, CAR-T 24, oncolytic virus therapy 25 and other immunotherapies 26. Thereby, other therapeutic modalities could be combined with immunotherapy targeting the PD-L1/PD-1 signaling axis to enhance the treatment outcomes by synergistic effect, exerting their complementary merits. It is noted that nanomedicine has been applied widely in targeting PD-L1 delivery for cancer immunotherapy due to their versatility and tunability, which can efficiently penetrate TME and specifically deliver to the major components in TME 27, 28. With unique physical properties and elaborate design, nanoparticles can be modified with targeting various molecules (e.g. anti-PD-L1 antibody) or loaded with various drugs (e.g. camptothecin), thus achieving nanosystems for the combination of immunotherapy and chemotherapy 29, even copackaging photosensitizer (e.g. pheophorbide) for synergistic cancer photoimmunotherapy 30.

In consideration of the huge advances in immunotherapy as an ongoing focus against tumors, here we review the various strategies to modulate the immune response through the PD-1/PD-L1 pathway. First, the structure, function and regulation of PD-L1 is introduced. Then, three main strategies targeting PD-L1 expression including PD-L1 blockade, downregulation of PD-L1and degradation of mature PD-L1, are severally discussed, which is based on different forms of PD-L1(e.g. membrane or cytoplasmic PD-L1) and various regulating mechanisms. Thereinto, FDA and National Medical Products Administration (NMPA) approved inhibitors or ongoing clinical trials drugs with PD-L1 regulation capacity is listed for exploration of other alternatives for targeting PD-L1 besides anti-PD-L1 monoclonal antibodies. Next, associated opportunities for cancer combination therapy are summarized, all of which aim to overcome the immunosuppressive environment of tumor cells and to maximize the benefits of immunotherapy. Moreover, application of nanomedicine in these strategies are noted and enumerated. At last, the limitations of immunotherapy targeting PD-L1 and future prospect of immunotherapy are presented. Overall, we aim to propose strategies that target all forms of PD-L1 expression at all levels, not only focusing on PD-L1 expressed on cell surface, which may be potential way to circumvent the obstacle of immune escape or immunosuppressive state.

## Structure, functions and regulation of PD-L1

**Figure 1:**
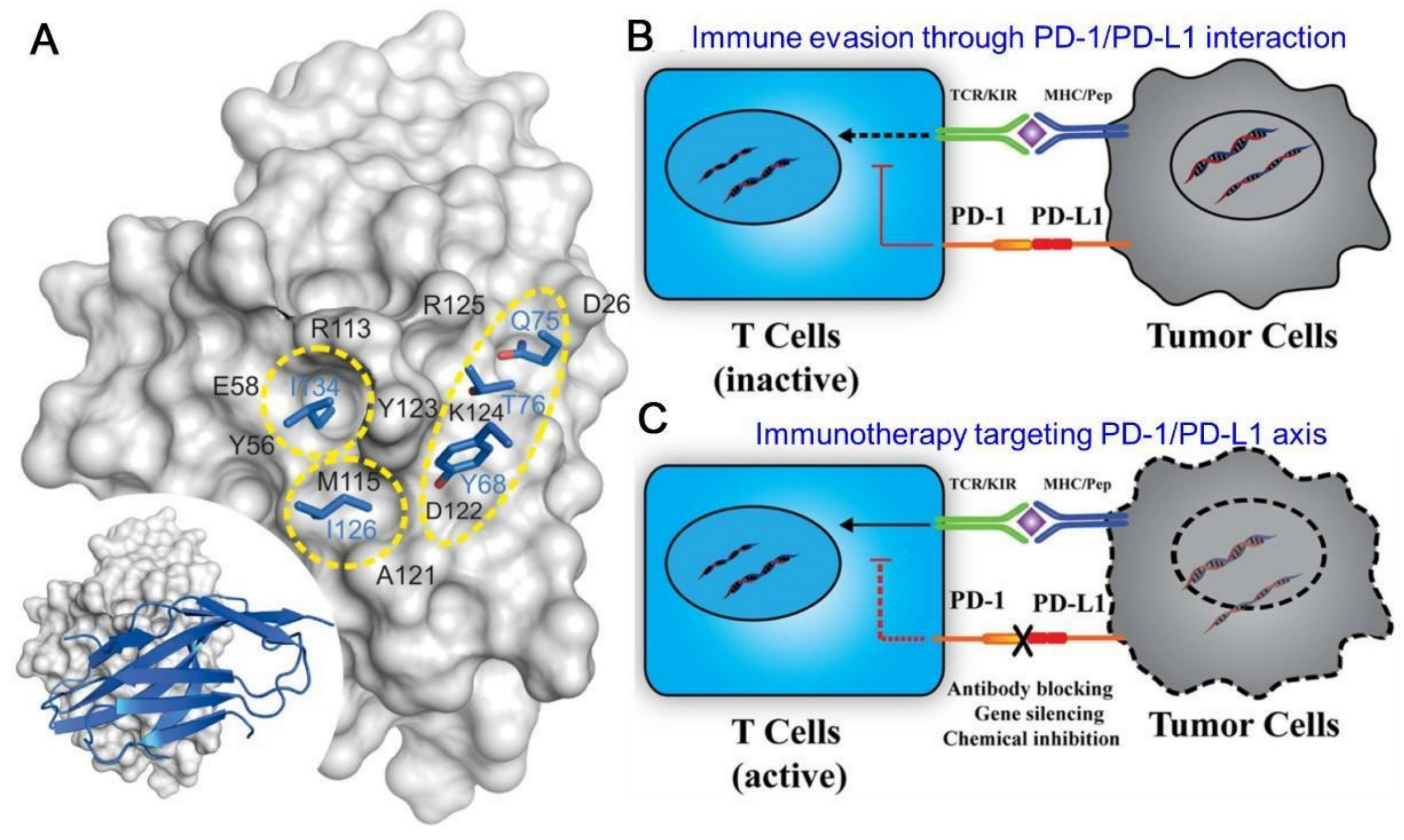
Schematic illustration of PD-L1 structure and the PD-1/PD-L1 engaged immune escape and immune therapy. (A) Three Main Hot Spots on the Human PD-L1 Surface. Residues from PD-1 are colored blue; PD-L1 is represented by gray surface, and the hot spots are marked by yellow circles. General orientation of the PD-1/PD-L1 complex is represented in the bottom left corner. Reproduced from Ref 34. Copyright 2015, Elsevier. (B) The binding of PD-1 to PD-L1 causes the tumor immune evasion, leading to the deactivation of T cells and survival of tumor cells. (C) The blocking of PD-1/PD-L1 reactivates the T cells and restores the immune response, resulting in the elimination of tumor cells. (B) and (C) are reproduced from Ref 184. Copyright 2019, Frontiers.

PD-1 (CD274) is a 40 kDa type I transmembrane protein, of which the expression can be detected on a wide range of cells including both hematopoietic cells and non-hematopoietic healthy tissue cells 9. The PD-L1 protein belongs to the immunoglobulin (Ig) superfamily, consisting of three compartments: an Ig-V and Ig-C-like extracellular domain, a transmembrane domain, and a short cytoplasmic domain 31-33. PD-1 (CD279) is a monomeric type I transmembrane proteins with characteristic Ig-variable (Ig V)-type topology attached to a transmembrane domain and a cytoplasmic domain with two tyrosine-based signaling motifs 34. Upon binding to PD-L1, PD-1 has a conformational change and can be phosphorylated at several tyrosine residues by Src family kinases, and thus can exert an inhibitory signal on activated T cells 35. The protein-protein interactions (PPIs) of PD-1/PD-L1 complex occurs through two large (1970 Å2) and flat binding surfaces that are structurally defined by several β-strand and β-turn motifs, which provides proof of principle of mimicry approach for peptide inhibitors designing 36. The molecular mechanics generalized Born surface area (MM/GBSA) analysis indicates that fragments N66-R86 and I126-E136 of PD-1 protein, and fragments I54-Y56 and R113-R125 of the PD-L1 protein are strongly involved in the interaction of both proteins 37. The human PD-L1 and human PD-1 were confirmed to form a 1:1 complex in stoichiometry in both crystal and solution state 34, 38. Three important hot spots have been identified in the interaction surface of human PD-L1 and PD-1, among which the I134 and I126 pocket can respectively interact with 6-membered aromatic ring and aliphatic hydrocarbons providing binding sites for consideration in drug development (**Figure 1A**) 39. Another ligand for PD-1 is PD-L2, which expresses mainly on DCs, macrophages and mast cells displaying more restricted expression 33, 40. The binding of PD-L1 or PD-L2 to PD-1 on the surface of lymphocytes could inhibit the peripheral immune response and prevent the immune storm, a critical way to maintain the immune self-tolerance. However, PD-L1 is also extensively detected in TME, and tumor cells smartly hijack this negative regulatory PD-1/PD-L1 pathway for immune evasion through the exhaustion and apoptosis of the infiltrating effector T cells (**Figure 1B**).

Earlier studies focused on the membrane PD-L1 (mPD-L1) as they directly interplay with PD-1, resulting in successful monoclonal antibody (mAb) inhibitors for anti-tumor immunotherapy (**Figure 1C**). Recent studies revealed that PD-L1 expressed on the surface of DCs also plays a key role in regulating antitumor immunity 41, 42. Deletion of PD-L1 in DCs, but not macrophages, greatly restricted tumor growth and led to enhanced antitumor CD8+ T-cell responses 41. It might be explained by the mechanism that blocking PD-L1 on the surface of DCs relieves B7.1 sequestration in cis by PD-L1, which allows the B7.1/CD28 interaction to enhance T cell priming 42. These suggest that PD-L1 blockade reinvigorates DC function to generate potent anticancer T cell immunity. In addition to mPD-L1, more forms of PD-L1 proteins have been observed, including nuclear PD-L1 (nPD-L1), cytoplasmic PD-L1 (cPD-L1), soluble PD-L1 (sPD-L1) and exosome surface PD-L1 (exoPD-L1). They are slightly different in structure, but also play important roles in the process of immunosuppression. nPD-L1 promotes tumor cell proliferation through chromatin remodeling process 43. cPD-L1 can be transported to the cell surface to replenish mPD-L1 recognized by antibodies, leading to the failure of antibody therapy 44. Moreover, cPD-L1 acts as an RNA-binding protein and completes with RNA exosome to increase the mRNA stability of DNA damage repair (DDR)-related genes and thus protects targeted RNAs from degradation, which boosts tumor resistance to DNA-damaging therapy 45-47. The specific function of sPD-L1 is unknown, but it has been detected in various cancers or inflammations 48. ExoPD-L1 inhibits the activity of lymphocytes, migrates to other PD-L1-negative tumors, and induces a systemic immunosuppressive state 49, 50. These discoveries not only provide possible explanations for the failure of traditional anti-PD-1/anti-PD-L1 therapy, but also present more targets and pathways for improved immunotherapy.

The regulating pathways of PD-L1 expression have been extensively studied and multiple mechanisms have been identified. Generally, PD-L1 expression can be regulated at the transcriptional, post-transcriptional, translational and post-translational levels in cancers 51, 52. Several transcription factors control *CD274* (coding PD-L1) transcription in response to various stresses or external stimuli (e.g. cytokines, hypoxia, epidermal growth factors). Interferon-γ (IFN-γ) is a major inducer of PD-L1 expression via the JAK-STAT-IRF1 signalling axis 53. Histone H3 lysine 4 trimethylation (H3K4me3) at the* CD274* promoter upregulates 54. while histone H3 lysine 27 trimethylation (H3K27me3) 55 suppresses the transcription of PD- L1 mRNA. RNA-binding proteins (e.g. ZFP36) [56]and a variety of microRNAs (e.g. miR-200c,) 57 modulates PD-L1 mRNA stability post-transcriptionally. At translational level, PD-L1 expression is primarily regulated via PI3K-AKT-mTOR and RAS-MEK-ERK (MAPK) pathways 58, 59. Several post-translational modifications, including phosphorylation 60, glycosylation 61, acetylation 62, ubiquitination 63 and palmitoylation 64 contribute to regulate PD- L1 degradation and activity.

## Anti-tumor strategies targeting PD-L1

On the basis of different forms of PD-L1 and various regulating mechanisms, we propose three strategies targeting PD-L1 for antitumor immunotherapy: (1) Blocking interaction of mPD-L and sPD-L1 with PD-1; (2) Downregulating all forms of PD-L1 production at transcriptional, post-transcriptional and translational level; (3) Degrading mature intracellular PD-L1 (cPD-L and nPD-L1) post-translationally. Comparing these three strategies, downregulation via gene silencing may have advantages over blockade and degradation of PD-L1, as a single interfering gene fragment is able to “switch off” the protein synthesis, fully stopping all forms of PD-L1 production and function. Downregulation by epigenetic mechanisms, microRNAs and 3' UTR variations have additional roles in fine-tuning the relative levels of PD-L1 in context-dependent settings 65. Degradation may target PD-L1 from the whole cell, minimizing the impact of redistribution of PD-L1 stock to the plasma membrane. Both downregulation and deregulation aim to reduce the overall PD-L1 reservoir.

As mentioned above, several mAbs have been approved by FDA or NMPA which target the PD-1/PD-L1 pathway, nevertheless, the clinical strategies only benefit a small fraction of tumor patients. As a result, other innovative methods are continuously explored in the PD-L1 immunotherapy. For example, besides the marketed aPD-L1 mAbs, exploration of other alternatives for targeting PD-L1 is ongoing, such as peptide, small-molecule drugs. For another instance, Metformin, an old drug widely used to treat type 2 diabetes, has been found to be a new drug as regulator of PD-L1 66, which provides a potential way for targeting PD-L1 immunotherapy. We listed the drugs with PD-L1 regulation capacity used in clinical and ongoing studies in Table 1, which can regulate PD-L1 by various mechanisms at different levels and be applied in the three strategies.

**Figure 2:**
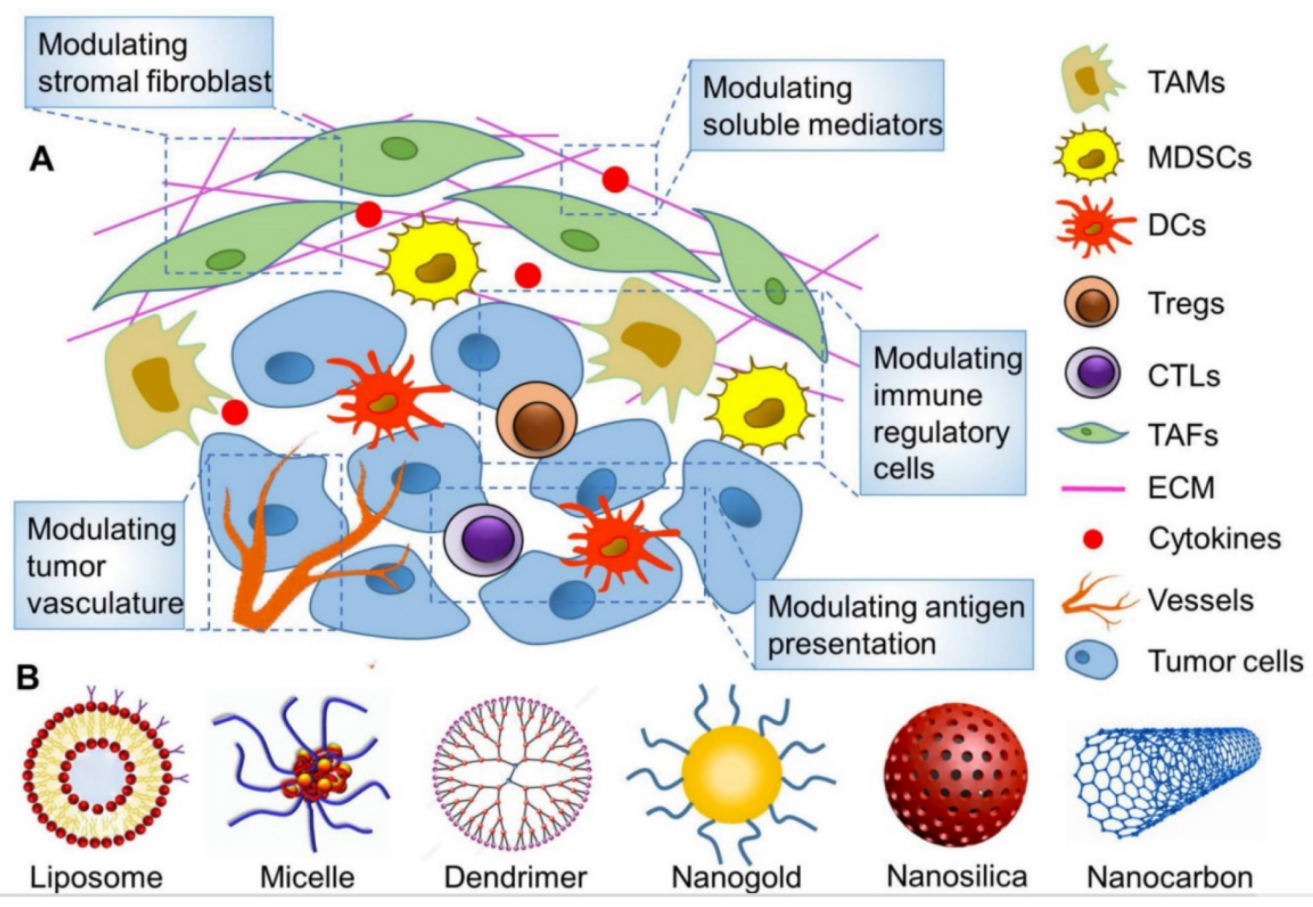
Targeted remodeling of immunosuppressive cells in the TME with nanoparticles to improve cancer immunotherapy. (A) The main strategies for modulating TME on the basis of nanoparticles. (B) Various reported nanoparticles used to improve cancer immunotherapy by remodeling TME. (A) and (B) were reproduced from Ref 28, copyright 2019, Ivyspring International Publisher.

We elaborate the above three strategies targeting PD-L1 as following that are committed to overcoming the existed obstacles and enhancing the therapeutic effect, hoping to inspire more ideas for immunotherapy. Thereinto, various types of nanoparticles have been used extensively to improve cancer immunotherapy by remodeling TME as illustrated in Figure 2 28. The nanoplatform can be loaded with different functional components to easily achieve combination therapy.

### Blockade of PD-L1

Blocking either PD-L1 or PD-1 can impede their binding, hence hindering T cells from receiving the immunosuppressive signal to maintain their activation and proliferation state, and then rendering T cells to perform the task of killing tumor cells. The commercial antibodies that can effectively bind to PD-L1 successfully verified this concept. Since the launch of the first PD-L1 inhibitor, atezolizumab, in 2016, there have been other four PD-L1 inhibitors (durvalumab, avelumab, envafolimab, and sugemalimab) listed in succession thereafter [Table 1]. It can be seen that the current clinically approved PD-1/PD-L1 inhibitors are only mAbs 67. However, conventional mAbs have several drawbacks, including restricted availability due to few patients responding, immune-related adverse effects (irAEs), poor penetration of tumor tissues, tedious and continuous administration, high price and vulnerability to drug resistance 67, 68. There are abundant binding sites on the surface of PD-L1, enabling the development of new antibodies and other forms of drugs to overcome the drawbacks.

#### Antibody

**Figure 3:**
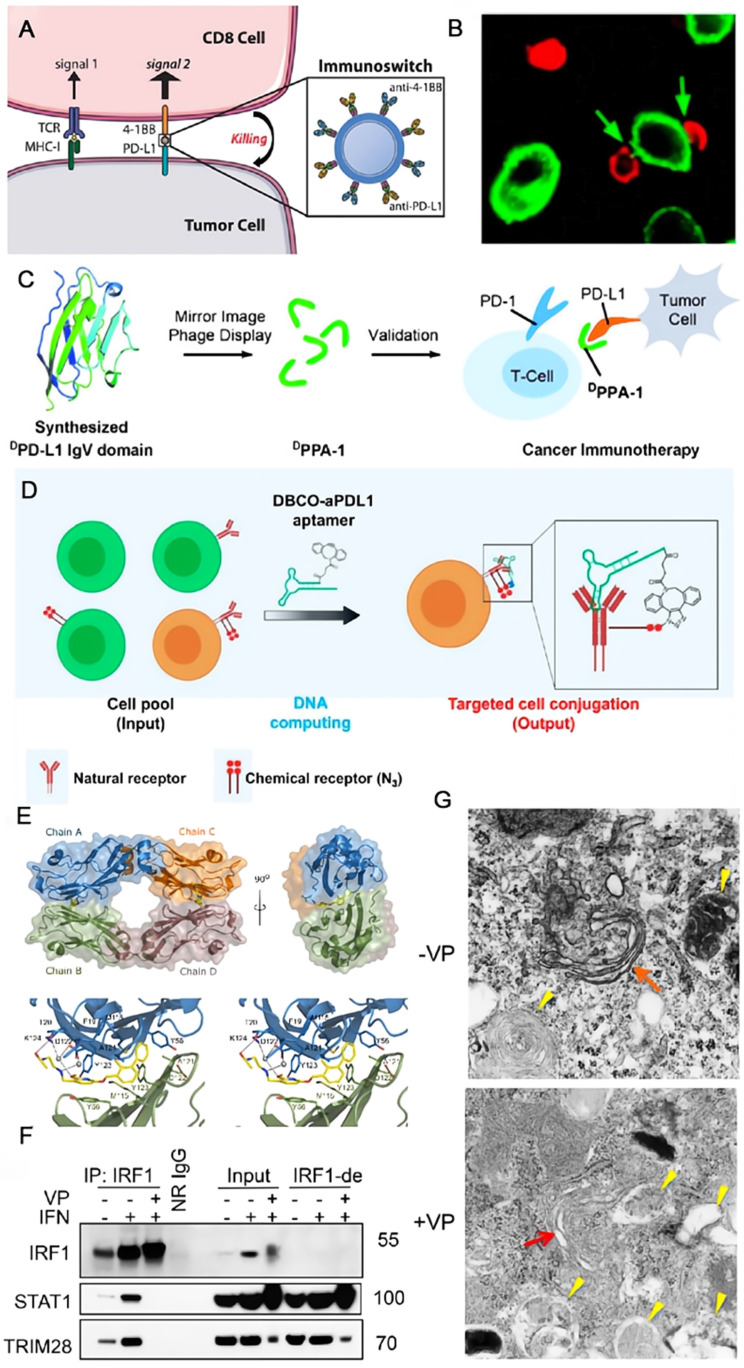
Schematic illustration of strategies to enhance the efficacy of immunotherapy through blockade of PD-L1. (A) A nano immunoswitch blocking both PD-L1 and 41BB concurrently enhances the immune response. (B) Increased binding was observed by confocal microscopy between 2C CD8+ cells (red) and B16-F10 cells (green) when mediated by the nano immunoswitch. (A) and (B) were reproduced from Ref 73. Copyright 2017, American Chemical Society. (C) A proteolysis-resistant D-peptide ^D^PPA-1 through protein chemical synthesis and mirror-image phage display, effectively blocks the interaction of PD-1/PD-L1. Reproduced from Ref 78. Copyright 2015, John Wiley and Sons. (D) The blocking of PD-L1 with aptamer-based logical computing reaction for precise and long-term immunotherapy. Reproduced from Ref 81. Copyright 2021, American Chemical Society. (E) Within the asymmetric unit four molecules of PD-L1 (mixed ribbon/ surface representation) are organized into two dimers (green and blue, and orange and brown). Each dimer binds a single molecule of BMS- 202 (yellow) at the dimer interface; Detailed interactions of BMS-202 at the binding cleft of PD-L1 dimer (stereoview). Reproduced from Ref 82. Copyright 2016, Impact Journals LLC. (F) Western blots for the level of proteins present in IRF1 immunoprecipitates (IP: IRF1) and mock immunoprecipitation with normal rabbit IgG (NR-IgG) from EFE184 cells treated with or without IFN-γ and verteporfin (VP) for 24 h. Blots for input and IRF1-immunodepleted (IRF1-de) lysates were shown on the right. (G) Autophagosomes as revealed by TEM (transmission electron microscopy) in EFE184 cells treated with (+) or without (-) verteporfin (VP, 1 μM) for 24 h. Red arrows, Golgi; yellow arrows, autophagosomes. (F) and (G) were reproduced from Ref 185. Copyright 2020, American Association for Cancer Research Inc.

To resolve the recurrence and low response of multiple myeloma to conventional antibodies, Ko et al. developed a novel antibody targeting PD-L1 based on a murine IgG subclass 2a 69. The anti-PD-L1 antibody (aPD-L1) can enhance the immune response of murine myeloma models by an additive effect via ICB and antibody-dependent cellular cytotoxicity (ADCC). Although mAbs are constantly being developed, they are still not effective against some cases of cancers. In this case, bispecific antibodies (bsAbs) that have two different antigen-binding sites in a single molecule are expected to play an important role 70. They can interact with two distinct surface antigens simultaneously, therefore, leading to the results of generating higher binding specificity, regulating two signaling pathways, or triggering the spatial connections of effector cells and target cells, which are supposed to be more efficacious and less toxic than the combination use of two mAbs 71. The bsAb YM101, developed by Wu et al., could target PD-L1 and transforming growth factor-beta (TGF-β) concurrently 72. TGF-β acts as a negative factor in advanced tumors that can decrease the cytotoxicity of T cells, inhibit the antigen presentation of DCs and facilitate the differentiation of Tregs. In the in vivo experiments, YM101 suppressed the adverse impacts of PD-L1 and TGF-β, and promoted a favorable TME. Schenck and coworkers conjugated two commercial antibodies, which are severally anti-PD-L1 for blocking the immune checkpoint on tumor cells and anti-41BB for enhancing the costimulatory signal on T cells, to the antibiotin-coated iron-dextran particles and obtained the injectable immunoswitch nanoparticles (**Figure 3A**). When mediated by these nanoparticles, the conjugation between effector and tumor cells increased clearly observed by the confocal microscopy (**Figure 3B**) 73. The nano immunoswitch displayed a superior effect on retarding tumor growth and prolonging survival in murine melanoma and colon cancer models by increasing the density and functionality of tumor-specific CD8+ T cells. Based on similar concept, Wang and coworkers developed a universal nanoparticle platform (αFc -NP) in which anti-IgG antibody (αFc) was grafted onto aminated nanoparticles through sequential Schiff base production and reduction reaction 74, 75. The advantage of this nano-adaptor is that it could employ either FDA-granted mAbs or those under clinical trials in this formula through Fc-specific noncovalent interactions. The augmented anti-tumor efficacy of the immunomodulating nano-adaptor has been verified in CD8+ T cell-, natural killer cell- and macrophage-mediated immunotherapy in various murine tumor models. As a nanomedicine, the accessibility of the ligand on nanostructure to the cell surface receptor is of significance for the therapeutic outcome. Greef et al. investigated the parameters that influence this recognition process using antibody functionalized DNA as a model taking advantage of the programmability of DNA origami 76. They found that the binding efficiency was primarily manipulated by the size of nanostructure and the location of DNA handle.

#### Peptide

Compared with the high price and immunogenicity of antibodies, the preparation of polypeptides is relatively simple with low cost, making it a promising candidate. Zhu and coworkers synthesized a peptide TPP-1 as the PD-L1 antagonist through bacterial surface display 77. TPP-1 was confirmed to specifically bind to PD-L1 with high affinity (*K_D_* = 0.095 μM) and inhibit the tumor growth, though its efficacy was not better than Durvalumab. Gao and coworkers screened the proteolysis-resistant ^D^PPA-1 for PD-L1 inhibition from a series of D-peptides using the cooperation of chemical synthesis and mirror-image phage display (**Figure 3C**) 78. ^D^PPA-1 is a low-molecular peptide with only 12 amino acid sequence of NYSKPTDRQYHF. It demonstrates a moderate *K_D_* value of 0.51 μM to PD-L1*in vitro*. However, the anti-tumor effect of ^D^PPA-1 was not satisfactory in the mouse model. They continued to screen and optimize the D-peptides, and obtained another stable one OPBP-1, showing much better anti-tumor capability 79. Given the nature of painless and favorable patient compliance of oral delivery, the authors loaded OPBP-1 to an oral delivery hydrogel with the aid of *N*, *N*, *N*-trimethylchitosan to maximize its bioavailability, and the yielded formulations displayed potent anti-tumor efficacy with minimal toxicity.

#### Aptamer

Aptamers are single-stranded nucleotide sequences which have specific binding ability to ligands but own deeper tissue penetration due to the small size (6-30 kDa). Just like antibodies, aptamers also show strong recognition ability and affinity to target molecules, so they are potential substitutes for antibodies. Yang and coworkers reported the first DNA aptamer targeting PD-L1 through a sequence of efficient pipeline 80. The aptPD-L1 could bind to PD-L1 and reinvigorate T cells with a series of cytokines elevated in TME, suppressing the tumor with negligible toxicity. The low objective response rate of anti-PD-1/anti-PD-L1 therapy is partially ascribed to the imprecise selectivity of mAbs, as normal cells also express PD-1 and PD-L1. Therefore, Tan and coworkers proposed an aptamer-based logic computing reaction to actualize precise targeting and long-lasting ICB (**Figure 3D**) 81. They attached dibenzocyclooctyne (DBCO) to the anti-PD-L1 aptamer and integrated azides into glycoproteins on the tumor cells. The recognition of aptamer along with the biorthogonal reaction-induced conjugation between DBCO and azides allows the standing retention of the aptamer without separation and internalization, hence increasing the anti-tumor efficacy with decreased side effects.

#### Small-molecule drugs

Small-molecule drugs that could be prepared by pure chemical synthesis allowing facile mass production show controllable cost, simple storage and deep penetration. While the development of small-molecule PD-L1 antagonist remains challenging, as the flat surface of PD-L1 make the drug design intricate. Holak and coworkers studied the structural basis of the nonpeptidic small-molecule BMS-202 and BMS-8 from a range of BMS compounds 82. BMS-202 can efficiently block the active surface of PD-L1 by inducing the dimerization of PD-L1. The detailed analysis of the crystal structure and the definition of the hotspots at the PD-L1 surface are supposed to inspire the exploration of new small-molecule PD-L1 inhibitors (**Figure 3E**). A design strategy of small molecule inhibitors for PD-L1 has been summarized by Wang and coworkers through analyses of the disclosed compounds 83. They noted that the pharmacophores normally contained four main parts: core group (a biphenyl moiety), linker (ether, alkene, or amide linkage), aryl group (aromatic ring or fused ring), and tail group (providing hydrogen binding). In addition to the specially synthesized drugs, Heimberger et al. utilized the commercial drug verteporfin for anti-tumor tests, and found that it can inhibit both the constitutive and reactive expression of PD-L1 84. Two mechanisms of action work together in the case of verteporfin and efficiently block the PD-L1 through the disturbed IRF1-STAT1 interaction **(Figure 3F)** and Golgi-related autophagy **(Figure 3G)**, suggesting that verteporfin regulates PD-L1 effectively through transcriptional and posttranslational mechanisms and represents an alternative therapeutic strategy for targeting PD-L1.

### Downregulation of PD-L1 expression

To reactivate the cytotoxic T cells and enhance their tumor killing ability, apart from PD-1/PD-L1 blockade, another feasible approach is to directly downregulate the expression of PD-L1, which would reduce the chance of interactions with its counterpart and diminish the intrinsic negative functions for anti-tumor immunity.

#### CRISPR/Cas9

**Figure 4:**
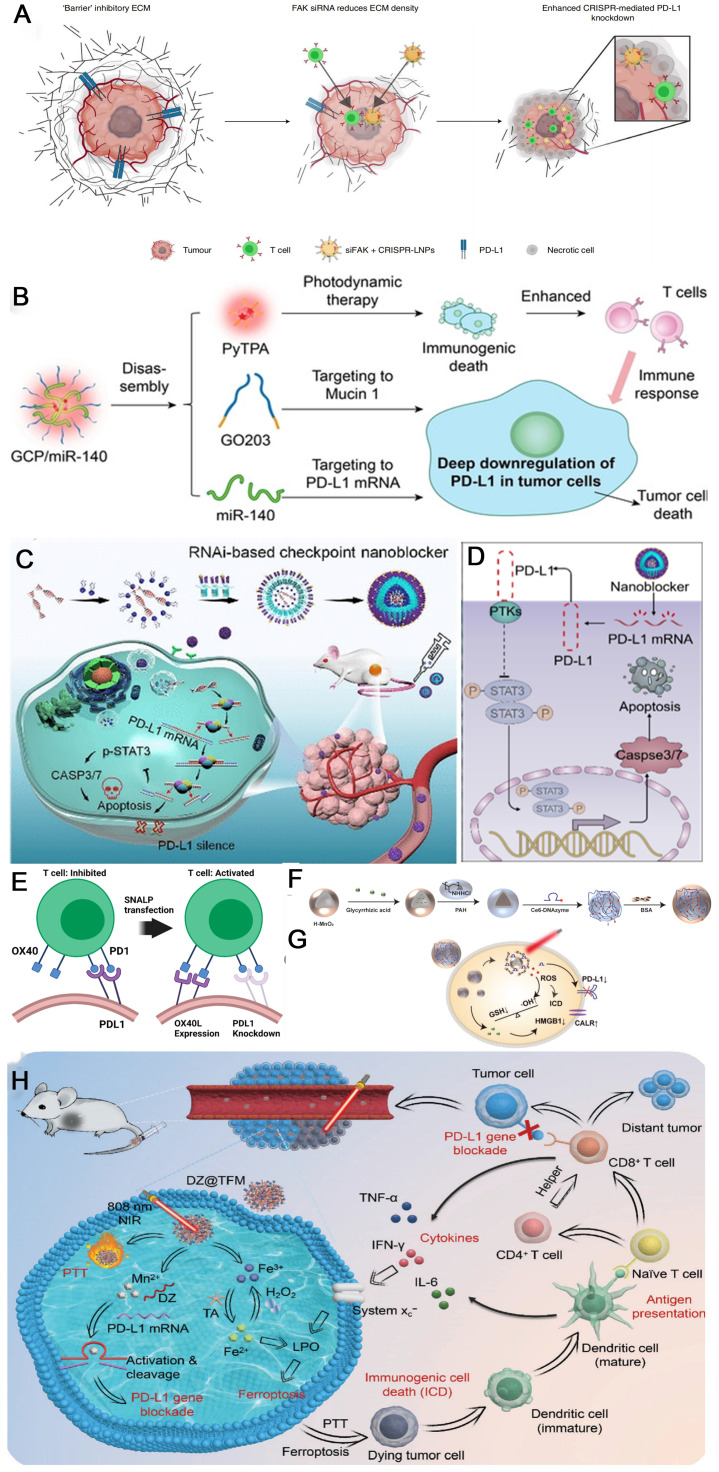
Schematic illustration of strategies to enhance the efficacy of immunotherapy through downregulation of PD-L1. (A) Schematic illustration for targeting the mechanical properties of tumors to open a double-checkpoint blockade of cancer (stiff ECM plus immunosuppression) to enable cancer therapy. Reproduced from Ref 89. Copyright 2022, Springer Nature. (B) Structure of GCP/miR-140, and the corresponding mechanism to deep downregulation of PD-L1. Reproduced from Ref 89. Copyright 2022, John Wiley and Sons. (C) Synthetic route of the siRNA-based PD-L1 nanoblocker and illustration about T cell-independent killing of cancer cells with the nanoblocker. (D) Proposed molecular mechanism associated with downregulation of PD-L1 expression and H460 cell apoptosis through the STAT3/caspase-dependent pathway. (C) and (D) were reproduced from Ref 91. Copyright 2020, American Chemical Society. (E) Stable nucleic acid-lipid nanoparticles reprogramming immune checkpoint interactions lead to inhibition of PD-L1 expression for cancer immunotherapy. Reproduced from Ref 92. Copyright 2020, Elsevier. (F) Synthesis schematic diagram of M-G/CDz@B nanosystem. (G) The role of M-G/CDz@B after entering the cells. (F) and (G) were reproduced from Ref 94. Copyright 2022, Wiley-VCH Verlag. (H) Schematic illustration of DZ@TFM for PTT-enhanced cyclically amplified tumor ferroptosis-immunotherapy. Reproduced from Ref 95. Copyright 2022, John Wiley and Sons.

Clustered regularly interspaced short palindromic repeats/CRISPR-associated nuclease9 (CRISPR/Cas9) gene editing technology, which can recognize the target genome sequence with single-strand guide RNA (sgRNA) and guide the Cas9 protease to knock down the target gene, has occupied a central position in further optimizing anti-PD-1/PD-L1 tumor immunotherapy 68, 85-87. For downregulating PD-L1 expression, the CRISPR/Cas9 technique can directly maximizes PD-L1 deletion at the genomic level, thus restoring function of lymphocytes in the TME. Siegwart et al. co-packaged focal adhesion kinase (FAK) siRNA, Cas9 mRNA and PD-L1 sgRNA into self-assembled lipid nanoparticles (LNPs) to resolve two critical barriers to cancer therapy (stiff extracellular matrix and PD-L1 overexpression) (**Figure 4A**). Gene editing was enhanced >10-fold in tumor spheroids due to increased cellular uptake and tumor penetration of nanoparticles mediated by FAK-knockdown. siFAK + CRISPR-PD-L1-LNPs significantly inhibited tumor growth and metastasis in four mouse models of cancer, including ovarian and liver cancer 86. Pallasch et al. observed that loss of TP53 led to decreased macrophage phagocytic capacity ex vivo, which is a frequent mechanism of resistance to chemoimmunotherapy (CIT) in B-cell malignancies. TP53 loss also increased PD-L1 expression and extracellular vesicles (EVs) formation by B-cell lymphoma cells. Disruption of EV bound PD-L1 by PD-L1 CRISPR-KO improved macrophage phagocytic capacity and in vivo therapy response 85.

#### MicroRNA

MicroRNA (miRNA) and small interfering RNA (siRNA) are two types of short-chain non-coding RNA that can modulate the gene expression by inhibiting mRNA translation or degrading mRNA via complementarily binding with the target mRNA. miR-424 (322) was sifted by Xu et al. from the miR-15 family which can reduce the PD-L1 expression by binding to the 3'-untranslated region (3'-UTR) of PD-L1 genes, hence, blocking the PD-1/PD-L1 axis 88. Overexpression of miR-424(322) reactivated the CD8+ T cells in the chemoresistance ovarian cancer cells through PD-L1 blockade way and reversed the chemoresistance state, allowing a synergistic therapeutic modality with chemotherapy and immunotherapy. miR-424(322) could also influence the CD80/CTLA-4 immune checkpoint and modulate the immune response. Lou and coworkers integrated three components containing caged GO203 peptide, miR-140 and an aggregation-induced emission luminogen (AIEgen) PyTPA into one nanoparticle GCP/miR-140 through self-assembling for enhanced immunotherapy by downregulation of the PD-L1 (**Figure 4B**) 89. Intracellular Cathepsin B disassembles the nanoparticles to three independent segments once they enter the cells. Then PD-L1 is deeply downregulated through two pathways: inhibiting the homodimerization process of mucin 1 by decaged GO203 peptide and preventing the translation process of PD-L1 mRNA by miR-140. Opposite to the miRNAs mentioned above, a series of miRNAs have been identified by Dong et al. that can upregulate the PD-L1 expression in cervical cancers 90, including miR-140, miR-142, miR-340, and miR-383, enabling new targets for PD-L1 related immunotherapy.

#### siRNA& shRNA

A RNAi-based nanocomposite containing a certain siRNA was constructed by Tan and coworkers for efficiently inhibiting the expression of PD-L1 (**Figure 4C**) 91. The nanoblocker demonstrated excellent stability and tumor-homing ability in the systemic circulation ascribed to the peptide-modified polymer outer shells. Molecular mechanism study reveals that the nanoblocker destroys the PD-L1 mRNA, which downregulates the p-STAT3 expression and intensely stimulates the activity of caspase 3/7, inducing tumor cell apoptosis (**Figure 4D**). In the NSCLC model, the nanoblocker knocked down over 60% of the PD-L1 expression and retarded the tumor growth powerfully. Importantly, this immunotherapy process does not require the engagement of toxic T cells. Aiming at a more efficient way to reprogram TME, mRNA (mOX40L) and siRNA (siPDL1) were simultaneously formulated into a single nanoparticle by Al-Jamal and coworkers (**Figure 4E**) 92. siPDL1 can downregulate the expression of the coinhibitory PD-L1, while mOX40L can upregulate the expression of the costimulatory OX40L, the synergy of which leads to much improved tumor immunogenicity.

Small hairpin RNA (shRNA) is always applied to generate a cell line with the stable knockdown or overexpression. Hao and coworkers used lentivirus-mediated PD-L1 shRNA to downregulate PD-L1 expression in leukemia cell-derived exosomes (LEXs) for optimizing LEX-based vaccine, which demonstrated a potential application in leukemia immunotherapy 93. Zheng et al. employed lentiviral plasmids carrying a shRNA targeting enhancer of zeste 2 polycomb repressive complex 2 subunit (EZH2) to regulate the expression of PD-L1 in hepatocellular carcinoma (HCC). The expression of PD-L1 was suppressed by epigenetic modificator EZH2 via directly upregulating the promoter H3K27me3 levels of *CD274* and* IRF1* in hepatoma cells 55.

#### DNAzyme

RNA cutting activity of deoxyribozyme (DNAzyme) is a type of metal iondependent DNA catalyst that is can function as gene regulation tool via silencing the expression of target mRNA, which was employed for the cleavage of PD-L1 mRNA to downregulate PD-L1 in tumor cells and subsequently avoid immune escape 94-96. Han and coworkers established a pH/glutathione responsive drug-loading hollow-manganese dioxide (H-MnO_2_)-based chlorine6 (Ce6)-modified DNAzyme bovine serum albumin (BSA) layer encapsulated therapeutic nanosystem (termed M-G/CDz@B) therapeutic nanosystem for the combination of gene therapy and immunotherapy (**Figure 4F**). Mn2+ generated by the degradation of nanoparticles via pH-induced hydrolyzation in the TME can be used as a catalyst for DNAzyme to cut PD-L1 mRNA. Auxiliary photosensitizers Ce6 can produce reactive oxygen species (ROS) under the irradiation of 660 nm near-infrared light, resulting in immunogenic cell death (ICD) (**Figure 4G**) 94. Similarly, Zhou and coworkers employed Tannic acid as ligand to form anti-PD-L1 DNAzyme (DZ) loaded photothermal Mn^2+^/Fe^3+^ hybrid metal-phenolic networks (MPNs) nanoplatform (termed DZ@TFM), in which Fe^2+^ is in situ generated from Fe^3+^ to trigger ferroptosis for inducing tumor cells ICD, while DNAzyme is activated by Mn^2+^ to effectively silence PD-L1 (**Figure 4H**) 95.

#### Peptide

RNA regulates the expression of PD-L1 from the perspective of gene modulation, which is intuitively a potent means, whereas other species have also been employed to achieve this purpose with individual advantages. The feasibility of peptide has been proved as shown in one of the prior examples where the peptide GO-203 can block mucin1 and downregulate the PD-L1 expression 89. C-terminal subunit of mucin 1 (MUC1-C) drives the expression of PD-L1 through occupying the NF-κB p65 on the PD-L1 promoter 97, and GO-203 as a polypeptide sequence with 16 amino acid sequences of RRRRRRRRRCQCRRKN, can bind to the CQCRRKN sequence on MUC1-C, hinder its homodimerization, and hence reduce the expression of MUC1-C and downregulate PD-L1 expression. In the allogeneic bone marrow transplantation model, the peptide antagonist (VIPhyb) of vasoactive intestinal peptide can inhibit the expression level of PD-1 and PD-L1, and enhance the anti-leukemia ability of CD8+ T cells 98.

#### Others

Some commercial drugs are also capable of downregulating PD-L1 expression and have attracted intense attention credited to their known toxicity profile. Berberine derived from *Coptis chinensis* has been shown to degrade PD-L1 by inhibiting the activity of CSN5 in NSCLC 99. Silibinin diminishes PD-L1 by reducing the activities of LDH-A and HIF-1α in nasopharyngeal carcinoma 100. Erianin inhibits HIF-1α and RAS and prevents their interactions, contributing to the downregulation of PD-L1 in HeLa cells 101.

### Degradation of mature PD-L1

**Figure 5:**
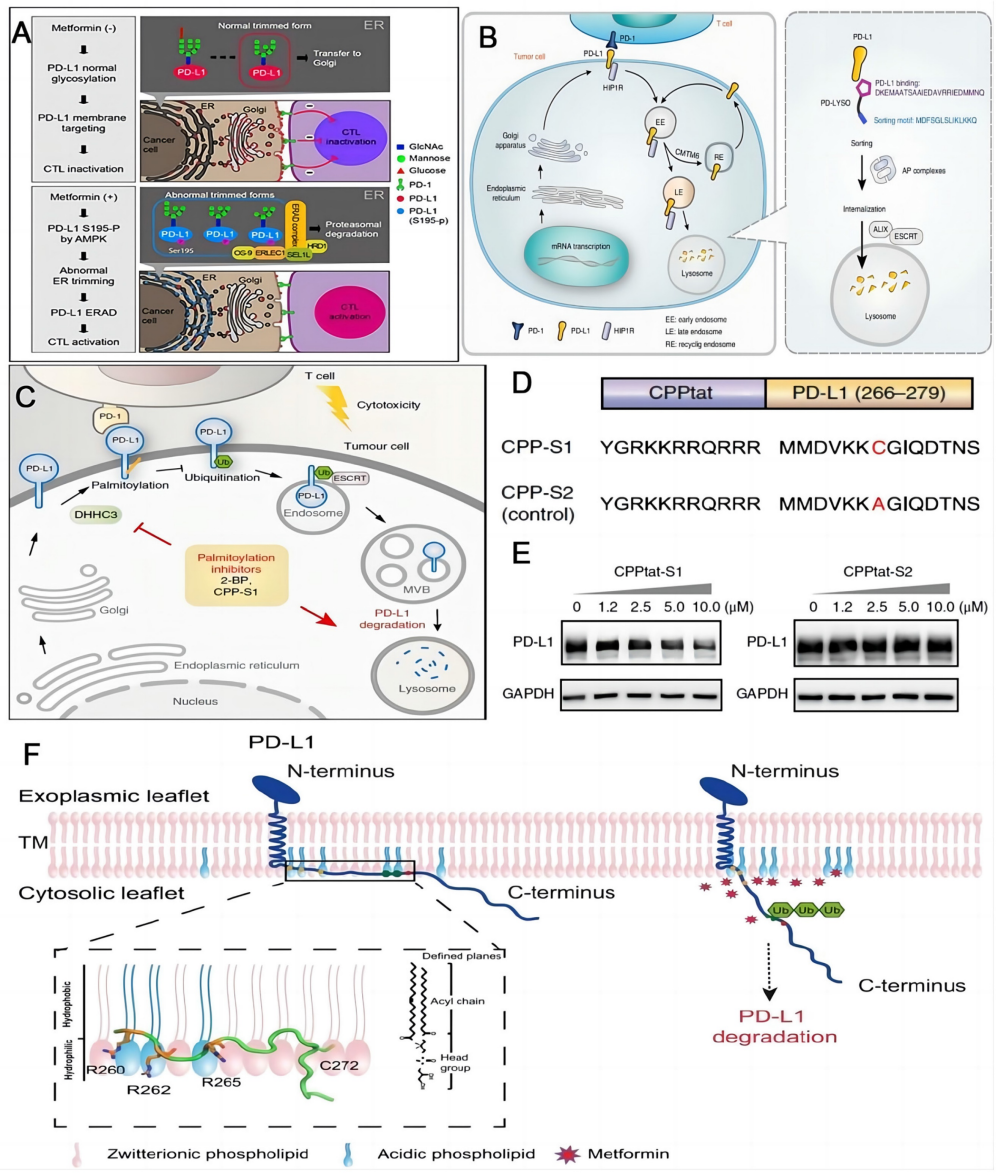
Schematic illustrations of strategies to enhance the efficacy of immunotherapy through degradation of PD-L1. (A) Schematic of the mechanism that metformin-activated AMPK phosphorylates PD-L1 at S195 to induce abnormal glycosylation and degrades PD-L1 through ERAD pathway. Reproduced from Ref 103. Copyright 2018, Cell Press. (B) PD-LYSO and HIP1R depleted PD-L1 through lysosomal degradation. Reproduced from Ref 110. Copyright 2022, Springer Nature. (C) Palmitoylation inhibitors facilitate PD-L1 ubiquitination and degradation. (D) Schematic of CPP-S1 and CPP-S2 peptides. The red highlights the differences between them. (E) Western blotting results of PD-L1 and GAPDH antibodies in RKO cells when incubated with CPPtat-S1 and CPPtat-S2 at different concentrations. (C), (D) and (E) were reproduced from Ref 44. Copyright 2022, Springer Nature. (F) Plasma membrane associated PD-L1 degradation through interruption of the electrostatic interaction between the polybasic residues and the acidic phospholipids. Reproduced form Ref 111. Copyright 2021, Springer Nature.

ICB with commercial mAbs is still facing challenges such as acquired resistance and adverse event. Degrading the checkpoint PD-L1 by lysosome, autophagosome and proteasome would alter the tumor immune tolerance and represents a promising alternative for PD-L1 inhibitors 102. Metformin was applied to intensify the activity of cytotoxic T lymphocyte by degrading PD-L1. Hung and coworkers firstly discovered that AMP-activated protein kinase (AMPK) triggered by metformin can straightly phosphorylate S195 of PD-L1, consequently giving rise to the aberrant PD-L1 glycosylation and endoplasmic reticulum associated degradation (ERAD) of PD-L1 (**Figure 5A**) 103. Based on the findings above, accumulating studies have reported the explorations for activation of AMPK to impair glycosylation and stabilization of PD-L1 to promote its degradation in tumor immunotherapy 104-109. Xu and coworkers designed a chimeric peptide using the sequences from Huntingtin interacting protein 1-related (HIP1R) based on the “binding-sorting” model for targeted lysosomal PD-L1 degradation (**Figure 5B**) 110. HIP1R is a natural regulator for lysosomal degradation of PD-L1. HIP1R (771-867) binds to PD-L1 intracellular region and HIP1R (966-979) mediates lysosome targeting and the following proteolysis. Xu's group also found that the palmitoylated form of PD-L1 in cytoplasmic domain stopped their ubiquitination process, consequently hindering the lysosomal degradation (**Figure 5C**) 44. On the contrary, the blockade of PD-L1 palmitoylation could facilitate the ubiquitination and lysosomal degradation, ultimately activating the anti-tumor immunity. This process can be realized by competitive inhibition of PD-L1 palmitoylation. The competitive inhibitor (CPP-S1) constructed by the cell-penetrating peptide (CPP) and the palmitoylation motif (S1 sequence) decreased the PD-L1 expression in a dose-dependent manner in RKO and LoVo cells, while there was no effect on the expression of PD-L1 when the control peptide (CPP-S2) was applied (**Figure 5D-E**). Either inhibition of the palmitoylation or deactivation of the related enzyme could activate the anti-tumor immunity by promoting the degradation of PD-L1. Focusing on the cytoplasmic part of PD-L1, OuYang and coworkers proposed that the electrostatic interactions between basic residues in the N-terminus of PD-L1 and acidic phospholipids of the plasma membrane control the cellular concentration of PD-L1 (**Figure 5F**) 111. Interruption of these interactions could enable the ubiquitination of PD-L1 and cause the PD-L1 degradation. Additionally, proteolysis targeting chimeras (PROTACs) technology, an emerging strategy to remove unwanted proteins by hijacking the ubiquitin-proteasome system 112, has been employed in enhancing the efficacy of PD-L1 degradation 113, 114. Cotton et al. developed an antibody-based PROTACs, fully recombinant bispecific antibodies that recruit membrane-bound E3 ligases for the degradation of cell-surface PD-L1 proteins 114. Chen and coworkers discovered novel resorcinol diphenyl ether-based PROTAC-like molecules as dual inhibitors and degraders of PD-L1 113.

Taking advantage of the overexpression of alkaline phosphatase (ALP) in TME, a series of compounds composed of a C-terminal peptide, an ALP-responsive trigger, a phenylalanine linker, and a capping moiety of adamantine have been reported by Yang's group 115.* Comp.3* screened from these materials, could self-assemble around PD-L1 on the cell membrane after reaction with ALP ascribed to their precisely engineered structure and subsequently cause the denaturation of PD-L1, indicative of an effective strategy for nanomedicine development. Similar to* Comp. 3*, a multiple component GlueTAC, which is consisted of a covalent nanobody, a lysosomal-sorting sequence and a cell penetrating peptide, could induce the internalization and degradation of membrane PD-L1 after the proximity-enabled covalent binding with PD-L1 116.

## Combination therapy

Although the immunotherapy targeting PD-1/PD-L1 axis is exciting and has gained great achievements in the past few years, low response rate, limited indications and considerable adverse events still frustrate the progress of mono-immunotherapy. Insufficient tumor immunogenicity, off-target toxicity and acquired resistance are all among the possible reasons for failed treatment. Therefore, the concept of combination therapy that takes advantage of other therapy modalities and remedies the shortcomings of immunotherapy has been put forward.

### Combined antibody therapy

Clinically, a combination of an ICI (atezolizumab, anti-PD-L1 mAb) with antiangiogenic therapy (bevacizumab, anti-vascular endothelial growth factor (VEGF) mAb) has led to major breakthroughs in treatment of metastatic NSCLC and renal cell carcinoma (RCC) 117, 118. More recently, Finn and colleagues reported that atezolizumab and bevacizumab combination therapy resulted in significantly longer overall and progression-free survival as well as strikingly better patient-reported outcomes than sorafenib (the antiangiogenesis multikinase inhibitor approved frontline systemic therapies for patients with unresectable HCC, which has become the new standard of care in patients with unresectable HCC 119, 120.The key molecular correlates of the combination therapy has been identified that anti-VEGF might synergize with anti-PD-L1 by targeting angiogenesis, Treg proliferation and myeloid cell inflammation 52.The overall response rate for melanoma using Nivolumab targeting PD-1/PD-L1 axis was only around 30% 121, and the soluble NKG2D ligands (sMIC) have been found to negatively correlate with the therapeutic outcome 10. Therefore, Wu and coworkers employed the anti-sMIC antibody (B10G5) to assist anti-PD-L1 mAb for melanoma mice treating, which turned out much longer survival compared with the monotherapy 122. The combination therapy augmented the therapeutic efficacy by activating splenic CD8 T cells, enhancing peripheral maintenance of NK cells and remodeling the TME. While in the study of Vitorino and coworkers on SCLC patients 123, mAb for PD-L1 was utilized as an auxiliary agent to maximize the benefit of rovalpituzumab tesirine, the clinical dosing of which is detrimental to patients though it has shown a high therapeutic efficacy. Sub-efficacious dose of rovalpituzumab tesirine with anti-PDL1 mAb successfully revealed persistent anti-tumor immunity through multiple signaling modulation pathways.

### Combined chemotherapy

**Figure 6:**
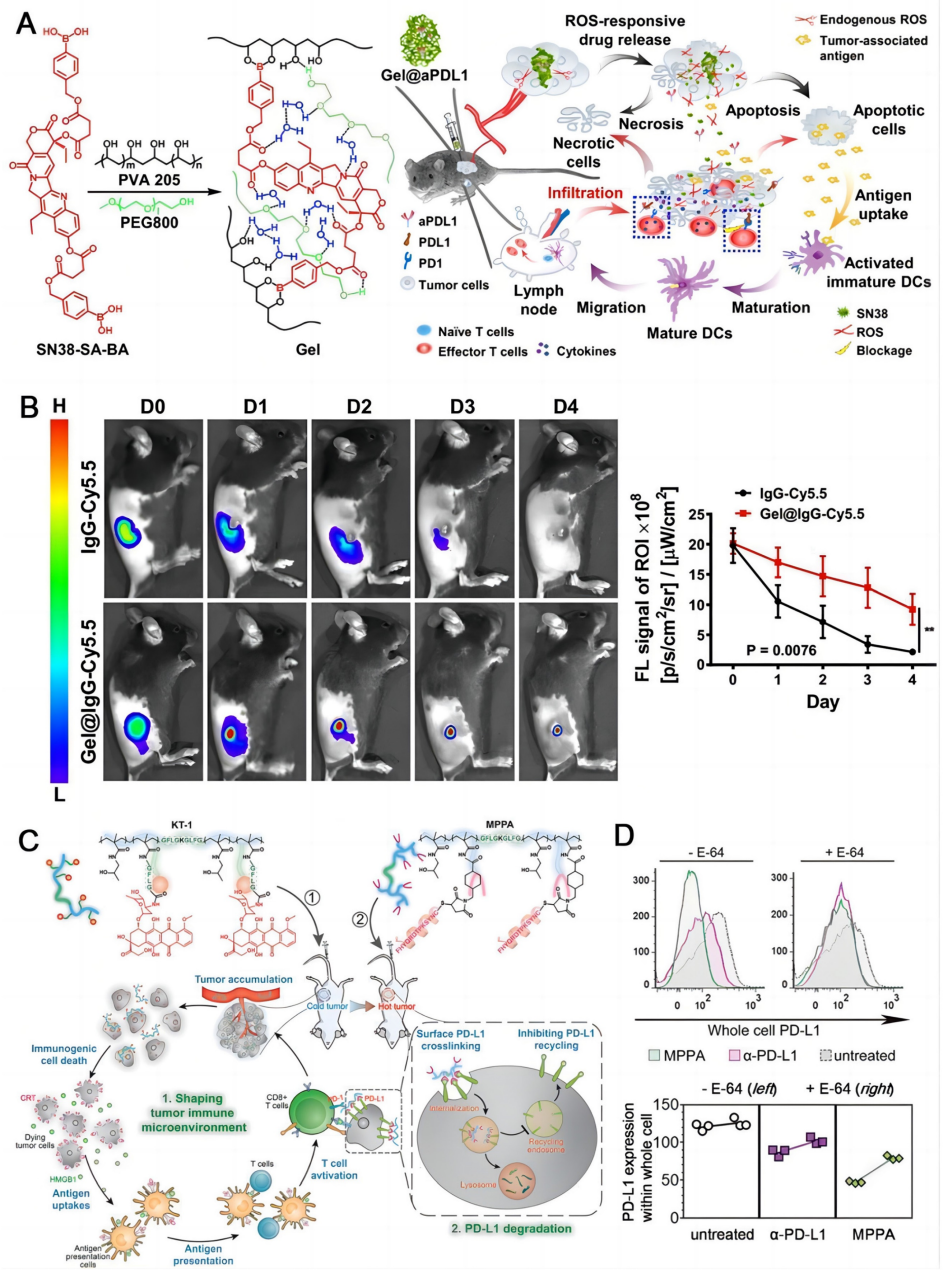
Chemotherapeutic drugs induce the immunogenic cell death. (A) Formation of the chemo prodrug PVA-SN38 hydrogel and the synergistic mechanism of action with aPD-L1. (B) Fluorescence imaging and semi-quantitative analysis of the free IgG-Cy5.5 and IgG-Cy5.5 loaded hydrogel in mice. (A) and (B) were reproduced from Ref 29. Copyright 2020, American Chemical Society. (C)Schematic illustration of Backbone-degradable HPMA copolymer facilitates tumor targeting of immunogenic drug to enhance its direct antitumor activity. (D) Whole cell PD-L1 expression with or without lysosome hydrolysis inhibition by E-64. (C) and (D) were reproduced from Ref 130, Copyright 2020, Wiley-VCH Verlag.

Chemotherapy is still a mainstream treatment modality for cancer, though it is normally not curative. Some classic chemotherapeutic drugs are able to induce the immunogenic cell death (ICD) besides directly destroying cancer cells, which holds great potential to activate the immune system 124-126. Chen and coworkers fabricated a compound agent (Gel@aPDL1) constructed by a ROS-responsive hydrogel (PVA-SN38) and an aPD-L1 (IgG) (**Figure 6A**) 29. Cyanine 5.5-labeled IgG (IgG-Cy5.5) as a surrogate for aPD-L1 was used to track the drug release process by confocal microscopy. The fluorescence of IgG-Cy5.5 loaded PVA-SN38 hydrogel decreased apparently slower than that of the free IgG-Cy5.5 in C57B6 mice, demonstrating a sustained and steady release of the antibody from the hydrogel (**Figure 6B**). After local injection, endogenous ROS degraded the hydrogel in TME, releasing SN38, a derivative of camptothecin for chemotherapy, and aPD-L1 for ICB. The synergistic effect of the two therapeutic modalities obviously inhibited the tumor growth in melanoma mice with no acute side effects. Nab-paclitaxel can stimulate the dying cells to release antigenic molecules which are helpful to immunotherapy 127. Pusztai et al. developed a combined dosing regimen using durvalumab targeting PD-L1 plus nab-paclitaxel and doxorubicin/cyclophosphamide for triple-negative breast cancer (TNBC) in stage I-III 128. The combination therapy demonstrated a pathologic complete response of 44%, which was positively associated with the PD-L1 expression. A highly cytotoxic drug MMAE was conjugated to Atezolizumab through a dipeptide linkage by Zhou and coworkers, and a bifunctional antibody drug conjugate (ADC) was obtained (ADC 3) 129. They discovered that Atezolizumab showed better endocytosis efficiency (50-60%) than Avelumab after interaction with PD-L1, which was a key factor for ADC design. ADC 3 demonstrated extremely potent cytotoxicity with EC_50_ of 9.75-11.94 nM in vitro and strong antitumor effect in vivo. Yang and coworkers employed polymer-based nanomedicines to develop two complexes, one was long-circulating epirubicin conjugate KT-1 for chemotherapy, the other was multivalent N-(2-hydroxypropyl) methacrylamide (HPMA) polymer-peptide antagonists to PD-L1 (MPPA) for PD-L1 degradation immunotherapy 130. KT-1 enhanced targeting of epirubicin while altering the tumor microenvironment in which PD-L1 expression was upregulated; Meanwhile increased cross-linking of PD-L1 by MPPA promotes its disassembly to pass through lysosomes, resulting in persistent inhibition (**Figure 6C**). The inhibition of intracellular PD-L1 by MPPA was stronger than that by aPD-L1 either at E-64 (**Figure 6D**), which is an irreversible, potent, and highly selective cysteine protease inhibitor that partially prevents enzymatic degradation in lysosomes 131.

### Combined radiotherapy

**Figure 7:**
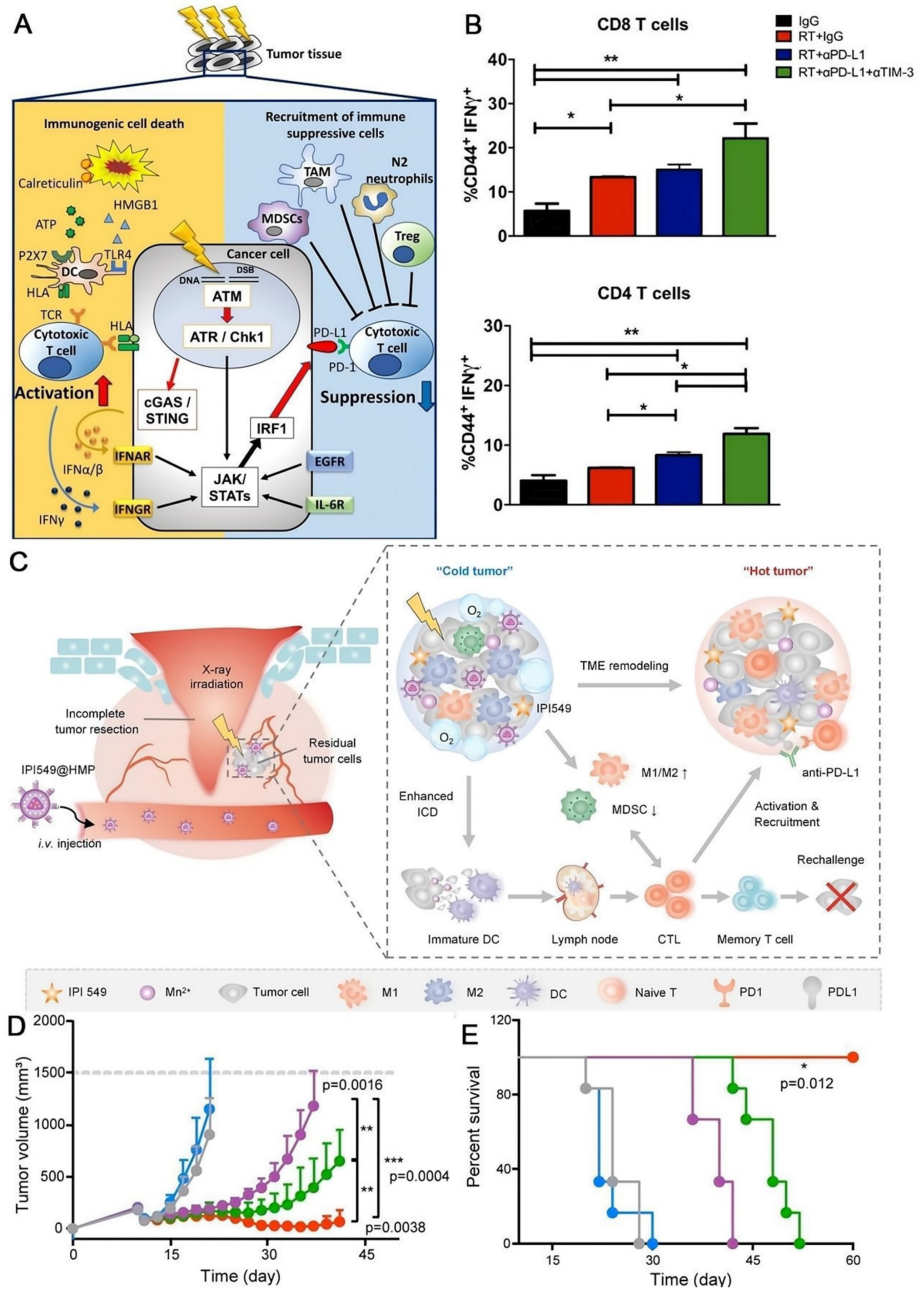
Antitumor effect of radiotherapy combined with immunotherapy. (A) Immune response induced by radiation therapy. Radiation therapy induces immunosuppressive expression of PD-L1 as well as immunostimulatory responses. Reproduced from Ref 132. Copyright 2020, Springer Singapore (B) Combined radiation therapy with PD-L1 blockade resulted in just transient effect in murine head and neck squamous cell carcinoma model. Flow cytometric analysis for T cell activation (CD44) and cytotoxicity (IFN-γ) in CD4 and CD8 T cells. (A) and (B) were reproduced from Ref 140. Copyright 2018, American Association for Cancer Research Inc. (C) Schematic illustrations of the nanomedicine IPI549@HMP combined with aPD-L1 for postsurgical tumor treatment. (D) Average tumor size and (E) survival rate of mice in each treatment group. (C), (D) and (E) were reproduced from Ref 140. Copyright 2022, Springer Nature.

Irradiation induces both immune stimulative and immune suppressive responses 132 (**Figure 7A**). Previous studies reported that PD-L1 expression was elevated simultaneously by radiation therapy 20, 133, 134. Therefore, radiotherapy can elicit host immunity by triggering ICD 135, releasing interferons 136, and upregulating the expression of MHC class I 137, enabling its combination with immunotherapy to provide a better prospect 138. While Karam et al. reported that the combined radiation therapy with PD-L1 blockade resulted in just transient effect in murine head and neck squamous cell carcinoma model, along with the resurgence of Tregs 139 (**Figure 7B**). So, they tried to deplete Tregs by anti-CD25 treatment in addition to the combination therapy, and thorough ablation of tumors were achieved, successfully overcoming the resistance issue. Surgery plays a pivotal role in solid tumor therapy; however, recurrence is still a great challenge leading to poor clinical outcomes. Unfortunately, the postsurgical TME is hypoxia and immunosuppressive, which severally correlates with the radiation resistance and immune response ineffectiveness, making the concept that using radiation therapy or immunotherapy to eradicate the undetected tumor nodules disabled. To tackle this conundrum, Yue and coworkers created a nanoplatform (IPI459@HMN) comprising hollow MnO_2_ (HMN) and PI3-Kinase γ inhibitor (IPI549) to boost the radiation efficacy (**Figure 7C**) 140. MnO_2_ catalyzes the overexpressed H_2_O_2_ in tumor milieu to O_2_, alleviating the hypoxia; and IPI549 reprograms the TME to immunogenic responsive, enhancing the immune response. Injection of IPI549@HMP combined with radiotherapy revealed the most pronounced inhibition of tumor outgrowth after surgery in Luc+ CT26 cancer model (**Figure 7D**) and no mouse died in this process (**Figure 7E**). When the augmented radiotherapy was further integrated with PD-L1 blockade, the combination therapy highly suppressed not only the locally residuals and metastatic tumors, but also tumor rechallenges.

### Combined photodynamic therapy

**Figure 8:**
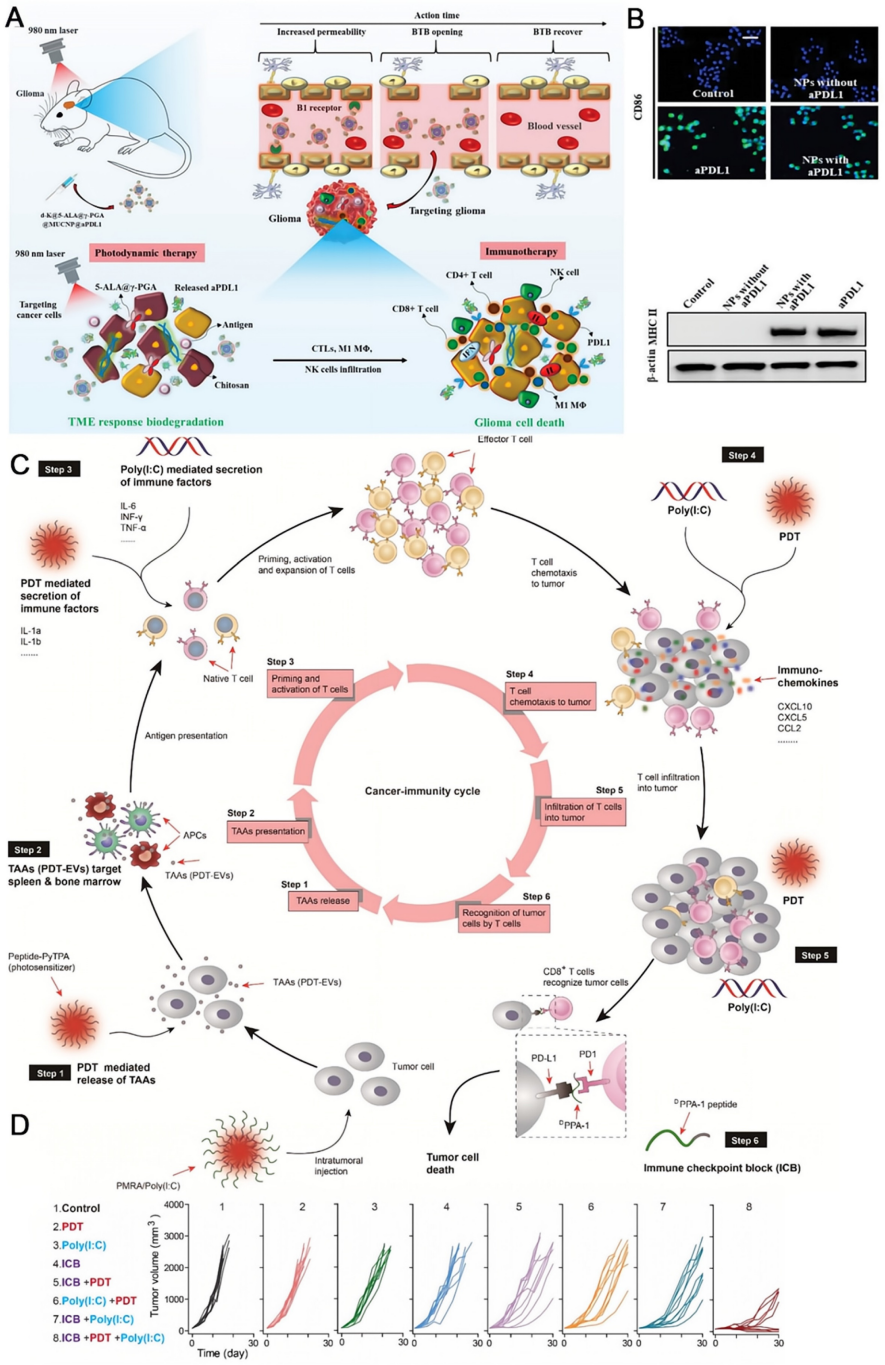
The combination of PDT and immunotherapy significantly suppressed the glioblastoma. (A) Schematic illustrations of antitumor immune responses induced by d-K @ γ-PGA @ 5-ALA @ MUCNP @ aPDL1-mediated PDT in combination with checkpoint-blockade. (B) MΦ were stained for stimulatory CD86 expression by the immunofluorescence assay after different treatments. And, the stimulatory MHC II expression was detected by WB assay. (A) and (B) were reproduced from Ref 145. Copyright 2021, Wiley-VCH Verlag. (C) Schematic illustrations of PMRA/Poly(1:C) mediated whole cancer immunity amplification for PDT assisted immunotherapy. (D) The growth inhibition of tumors in different treatment groups. (C) and (D) were reproduced from Ref 146. Copyright 2022, Elsevier BV.

Photodynamic therapy (PDT) that employs reactive oxygen species (ROS, such ^1^O_2_, •OH) generated by photosensitizers under light irradiation to kill tumor cells are widely studied ascribed to non-invasiveness, precise treatment and low resistance 141-144. Brain cancers frequently badly responds to immunotherapy, due to the factors including blood tumor barrier, immune privilege of central nervous system, and immunosuppressive TME. Sun and coworkers developed a biodegradable polymeric nanoparticle with multiple components comprising a commercial photosensitizer 5-ALA, a PD-L1 antibody, and upconverting nanoparticles, which was further coated by B1R kinin ligand to assist crossing the blood tumor barrier (**Figure 8A**) 145. The self-assembled nanomedicine dissociated at the tumor site. Upon illumination with a 980 nm laser that is located in the tissue transparence window, visible light mediated PDT was implemented with the help of upconverting nanoparticles, not only causing the apoptosis and necrosis of cancer cells, but also altering TME and changing the immunogenic “cold” environment to a “hot” one, including activating tumoricidal macrophages (**Figure 8B**). Then, the released aPD-L1 could efficiently activate T cells to eliminate tumor cells. The combination of PDT and immunotherapy here significantly suppressed the glioblastoma. Lou and coworkers designed a nanocomposite self-assembled by a modular peptide PMRA and poly(I:C) to realize the improved immunotherapy with the aid of PDT (**Figure 8C**) 146. PMRA is conjugated by a PD-L1 blocking peptide ^D^PPA-1, an MMP-2 cleavage site, a poly(I:C) loading peptide R, and an AIEgen photosensitizer. Active species are released in the tumor site where the nanocomposite is broken down by MMP-2. PDT mediated by the AIEgen facilitates the production of tumor-associated antigens (TAAs), which triggers the priming of T lymphocytes. The chemokines from PDT and Poly(I:C) also help to activate T lymphocytes. Consequently, the anti-PD-L1 therapy with ^D^PPA-1 could efficiently kill tumor cells in the existence of T lymphocytes. Importantly, the ICD gives rise to more TAAs that would enter the therapeutic cycle, resulting in a cascade amplification effect. As shown in **Figure 8D**, triple-therapy with ICB +PDT +Poly(I:C) using PMRA/Poly(I:C) displayed the best tumor retarding effect in B16-F10 xenograft tumor models with an objective response rate of near 100%, compared with the mono- and dual-therapy modalities. Further studies confirmed that this synergistic treatment mode not only impeded the growth of subcutaneous tumors, but also eliminated the lung metastasis by enhancing the whole immunity cycle.

### Combined photothermal therapy

**Figure 9:**
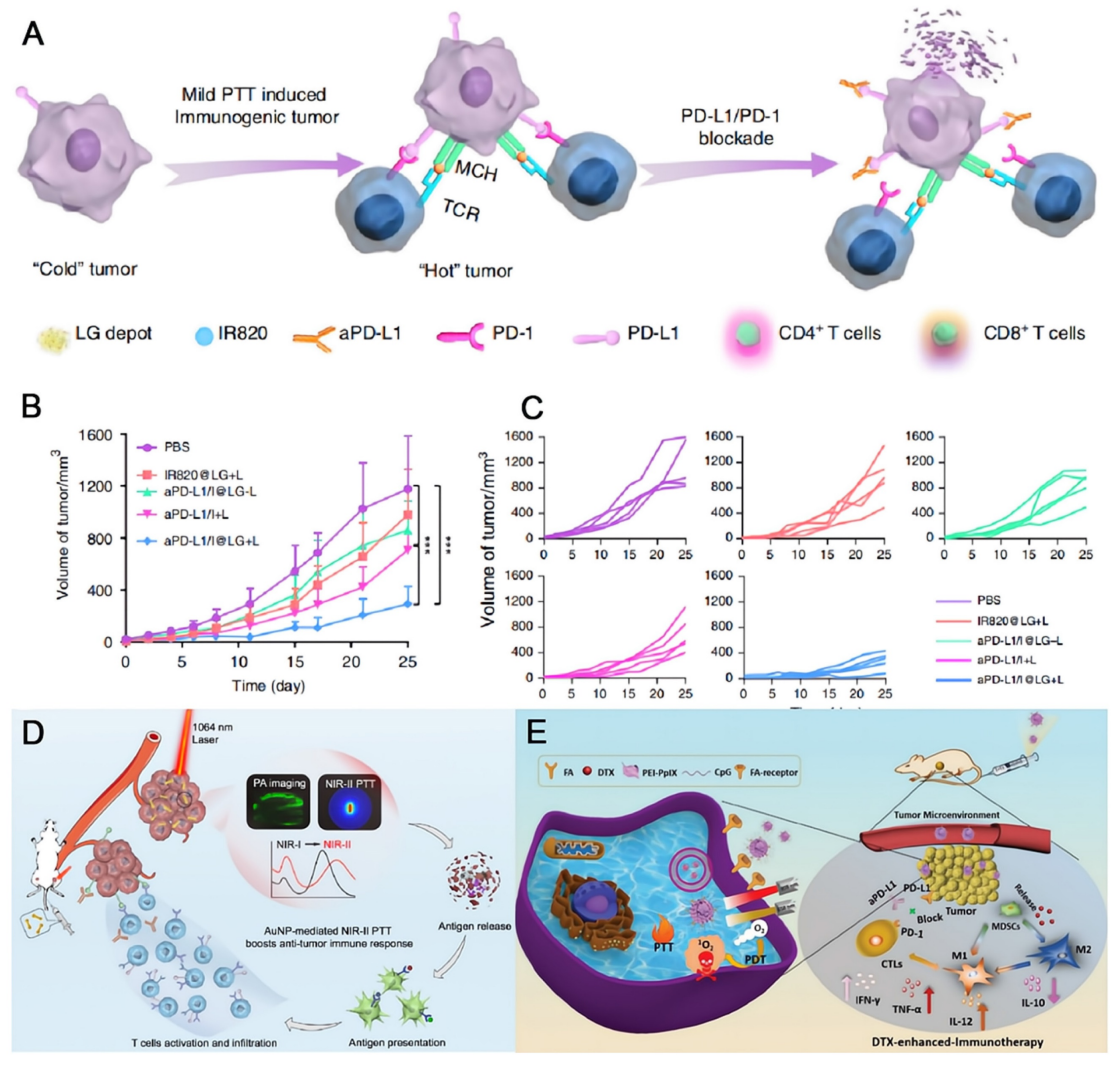
The therapeutic effect on tumors with symbiotic mild photothermal-assisted immunotherapy (SMPAI) strategy. (A) Schematic illustrations of transforming a “cold” tumor to a “hot” one by aPD-L1/I@LG. (B) Changes of mean distal tumor sizes of mice in different treatment groups. (C) Distal tumor growth curve of individual mouse in different groups. (A), (B) and (C) were reproduced from Ref 151. Copyright 2019, Springer Nature. (D) Schematic illustration of utilizing AuND to achieve NIR-II PTT facilitated cancer immunotherapy. Reproduced from Ref 151. Copyright 2020, Elsevier. (E) Schematic illustrations of design and synthesis of FA-CD@PP-CpG nanocomposites, and illustration of FA-CD@PP-CpG for docetaxel-enhanced immunotherapy. Reproduced from Ref 158 Copyright 2019, Wiley-Blackwell.

Photothermal therapy (PTT) relies on hyperthermia to kill cancer cells with or without contrast agent upon external light irradiation 147, 148. It also shows the advantages of noninvasiveness and controllable properties similar to those of PDT. The tumor ablation ability is highly associated with the temperature that PTT induces. High temperature over 50 °C quickly eliminate the tumor tissues, while the normal ones are not spared 149. Mild temperature around 45 °C is much less destructive, but the damage could be repaired by heat-shock proteins 150. However, even a low temperature elevation could remodel TME, allowing the combination of PTT and immunotherapy a promising way to overcome their individual drawbacks. Sun et al. designed a lipid gel (LG) depot encapsulating a photothermal agent and an aPD-L1 for symbiotic mild photothermal-sensitized immunotherapy (SMPAI) 151. Upon light irradiation, a mild PTT takes place, which upregulates the PD-L1 on tumor cells and increases the tumor infiltrating lymphocytes (TILs). Such a reprogrammed immunogenic TME is beneficial for aPD-L1, which is controllably released concurrently due to gel-to-sol transformation triggered by the small temperature ascent (**Figure 9A**). Using IR820 (I) as the photothermal agent, the LG depot (aPD-L1/I@LG) showed the most significant tumor inhibition effect under NIR light irradiation in 4T1-tumor-bearing BALB/c mice which had insufficient immunogenicity. When the distal tumor models were built along with the primary tumor models, the localized treatment on the primary tumors with aPD-L1/I@LG under light irradiation also displayed the best therapeutic efficacy on the distal tumors (**Figure 9B-C**), indicating the controllable drug release, long-term antitumor effect, and efficient abscopal immune response of the SMPAI strategy. Regarding to phototherapy, the therapeutic effect is largely limited by the penetration depth of incident light. The contrast agent that can be excited by photon in the second near-infrared window (NIR-II) is highly desirable, ascribed to minimal light scattering and improved penetrance as deep as 3~5 cm 152, 153. Gold nanodumbbells (AuNDs) were fabricated by Huang and coworkers that can be excited by a 1064 nm laser with a high photothermal conversion efficiency of 84.9% 154. The localized surface plasmonic resonance peaks of gold nanoparticles are finely tuned by DNA-programming strategy. Postsurgical NIR-II PTT with the nanotheranostics plus anti-PD-L1 therapy efficaciously suppress the metastatic tumor cells (**Figure 9D**).

As just noted, chemotherapy, radiation therapy, PDT, and PTT all have demonstrated the capacity to alter TME, ameliorating the immune response of an immunologically “cold” tumor, therefore, an enhanced tumor ablation could be expected in combination with PD-L1 blocking therapy. In addition to such two-prong strategy, more than two therapeutic modalities have also been integrated, hoping to result in better anti-tumor effects 155-157. Dong and coworkers designed a multicomponent nanocomposite for targeted tumor treatment in the synergistic cooperation of PDT, PTT, chemotherapy, and immunotherapy (**Figure 9E**) 158. Briefly, docetaxel is loaded by mesoporous CuS followed by the surface modification of tumor target ligand folic acid. Then the final nanocomposite FA-CD@PP-CpG is obtained by coating the prior nanoparticle with alternative PEI-PpIX and CpG. Thereinto, docetaxel can improve the immune response of tumor cells by reducing myeloid-derived suppressor cells and inducing the M1-like phenotype polarization; CuS shows the photothermal conversion ability; PpIX is a photosensitizer that can execute PDT; and CpG as an immunoadjuvant can stimulate immune response and release proinflammatory cytokines. When combined with aPD-L1, the nanomedicine displays potent anti-tumor efficacy upon dual-laser irradiation under 650 and 680 nm in breast cancer mouse model.

## Summary and outlook

Here we summarize the current status and dilemmas of PD-L1-related immunotherapy. In fact, ICB has indeed achieved remarkable success in a wide range of tumor diseases, and become a frontline treatment in anti-tumor therapy. However, the objective response rate of ICB is still not satisfactory. As a result, the improved treatment strategy is highly desirable. In this paper, we propose three strategies targeting PD-L1 based on different forms of PD-L1 and various regulating mechanisms to enhance the therapeutic effect, including blockade of the interaction between PD-L1 and PD-1, downregulation of PD-L1 expression and degradation of mature PD-L1. Thereinto, we describe a variety of materials have been designed to target PD-L1 regulation, including antibodies, nanoparticle, peptide, aptamer, RNA, and small molecule agents. Additionally, we list the drugs with PD-L1 regulation capacity used in clinical and ongoing studies to explore other alternatives for targeting PD-L1 besides anti-PD-L1 monoclonal antibodies. Moreover, we discuss associated opportunities for cancer combination therapy with other modalities such as chemotherapy, radiotherapy, PDT and PTT, as these conventional or emerging modalities are capable of increasing the immune response of tumor cells by altering TME, and would display synergistic effect.

Despite great achievements have been made in improving the efficacy of immunotherapy, a series of problems still remain. Firstly, although anti-PD-1/PD-L1 therapies exhibit potent antitumor effects, only patients with certain cancer subtypes can benefit from anti-PD-1/PD-L1 immunotherapy, such as NSCLC 159, TNBC [160]and melanoma 161. It may because high levels of PD-L1 expressed on cancer cells and/or in TME in these types of cancer and therefore respond well to immunotherapy, which works by blocking the interaction between PD-L1 and PD-1 on T-cells. But these data are contrary to the results in squamous NSCLC, PD-L1 expression does not affect the clinical activity and safety of Nivolumab 160. This suggests that the use of PD-1/PD-L1 as the sole predictive biomarker for tumor immunotherapy remains problematic and how to identify patients who may benefit from the anti-PD-1/anti-PD-L1therapy is a critical clinic bottleneck. Secondly, so far, all of the approved inhibitors of PD-l or PD-L1 are mAbs. However, the drawbacks of mAbs, such as low response rate, irAEs, acquired resistance, poor permeability to tumor tissue, always discontinue the treatment and thus limit the clinical efficacy of ICB therapy. It urgently requires development of potential therapeutic strategies to circumvent disadvantages of conventional anti-PD-1/PD-L1 antibodies. Thirdly, the feasibility and systematic toxicity of nanomedicine should be further carefully evaluated. Nanocomposite seems to arouse intensive attentions in combination therapy as it is easy to integrate diverse functional components into a single formulation. However, apart from the efficacy, other factors that may impede their clinic translation should be concerned, such as the reproducibility of the nanoparticle, the cost-benefit analysis, the toxicity, the standardized medication, etc. Finally, most of the studies are limited to animal models, especially mouse models. There are large differences between humans and animals, and the strategies that show excellent therapeutic effects on animals probably do not work in humans. For example, it only takes a few weeks to form a tumor mouse model, while a few years are necessary for a human to develop the same kind of tumor.

In short, we aim to propose strategies targeting PD-L1 expression and associated opportunities for cancer combination here and also hope more drugs and nanocomposites as well as antibodies would be achieved clinical translation to address the bottlenecks and maximize the advantages of immunotherapy.

## Figures and Tables

**Table 1:** List of FDA/NMPA approved mAbs or ongoing clinical trials drugs that regulate PD-L1

Drug	Targets	Indication	Strategy	Function pathway	Refs
Atezolizumab (FDA and NMPA)	PD-L1	Urothelial carcinoma, NSCLC, TNBC, SCLC,HCC	Blockade	prevents the interaction of PD-L1 with PD-1 or B7.1, does not affect the interaction between PD-L2 and PD-1	117
Avelumab (FDA and NMPA)	PD-L1	Advanced RCC,Advanced or metastatic UC	Blockade	inhibits the interaction between PD-1 and PD-L1, leaving PD-1/PD-L2 interactions intact; induce ADCC of tumor cells	162
Durvalumab (FDA and NMPA)	PD-L1	Urothelial carcinoma, NSCLC, SCLC	Blockade	completely blocks the binding of PD-L1 to both PD-1 and CD80	163
Envafolimab (NMPA)	PD-L1	Advanced dMMR/MSI-H solid tumorsBlocked	Blockade	inhibits the interaction of PD-L1 with PD-1 or CD80, does not induce ADCC and complement dependent cytotoxicity (CDC).	164
Sugemalimab (NMPA)	PD-L1	Metastatic NSCLC; Locally advanced, unresectable, stage III NSCLC	Blockade	inhibits the interaction of PD-L1 with PD-1 or CD80, does not induce ADCC and CDC.	165
GO-203-2c	Mucin1	Solid Tumors	Downregulation	blocks mucin1 and reduces the PD-L1 expression	166
Metformin	AMPK (activation)	Multiple cancers	Degradation	increases phosphorylation and degradation of PD-L1	66
CA-170	PD-L1	Multiple cancers	Blockade	binds to PD-L1 and inhibits PD-1 signalling; disrupts the interaction between PD-1 and PD-L1;	167
Gefitinib, erlotinib and osimertinib	EGFR	NSCLC, HNSCC and TNBC	Downregulation and degradation	downregulates PD-L1 transcription owing to decreased JAK-STAT, PI3K-AKT and/or MAPK signalling; activates GSK3α and GSK3β to increase phosphorylation, ubiquitination and proteasomal degradation of PD-L1	168, 169
Various inhibitors	PI3K-AKT-mTOR pathway	Multiple cancers	Downregulation	downregulates PD-L1 transcription owing to decreased AP1, STAT3, YAP1-TAZ and β-catenin signalling; downregulates PD-L1 mRNA translation	170, 171
Various BRAF or MEK inhibitors	RAS-RAF-MEK-ERK pathway	Multiple cancers	Downregulation	downregulates PD-L1 transcription owing to decreased AP1 and STAT3 signalling; increased mRNA decay activator protein ZFP36 (tristetraprolin)- mediated PD-L1 mRNA degradation; however, in certain contexts, inhibition of RAF-MEK upregulates PD-L1 or IFN-γ-induced PD-L1 via unknown mechanisms	172, 173
Tofacitinib, ruxolitinib and fedratinib	JAK	Nasopharyngeal carcinoma,TNBC and NSCLC	Downregulation	inhibits STAT1/3-mediated transcription to downregulate PD-L1	174, 175
Verteporfin	STAT1	Melanoma	Degradation	inhibits STAT1-IRF1 interaction and thus PD-L1 transcription and also promotes autophagic degradation of PD-L1	176
Various direct and indirect YAP1/TAZ inhibitors	YAP1/TAZ signalling	Multiple cancers	Downregulation	downregulates YAP1/TAZ-mediated transcription of PD-L1	176, 177
LGK-974,MK-2206 and etoposide	Various -WNT-β- catenin Pathway- components	Multiple cancers	Downregulation	the Porcupine inhibitor LGK-974 downregulates PD-L1 expression in TNBC cells via an unknown mechanism; the AKT inhibitor MK-2206 suppresses β-catenin activation and thus transcriptionally downregulates PD-L1 in glioblastoma cells; etoposide suppresses β-catenin signalling and thereby transcriptionally downregulates the N-glycosyltransferase STT3, resulting in decreased glycosylation and thus destabilization of PD-L1	171, 178
Doxorubicin	ZFP36	NSCLC and breast cancers	Downregulation	downregulates PD-L1 translation by indirectly activating ZFP36, which binds to and destabilizes PD-L1 mRNA	179
Tomivosertib	MNK1/2	NSCLC, breast cancers and Castrate-resistant Prostate Cancer	Downregulation	suppresses eIF4E phosphorylation to downregulation PD-L1 mRNA translation	180
BAY1895344 and VE-822	ATR	Prostate cancer and TNBC	Degradation	activates CDK1-SPOP mediated degradation to downregulates PD-L1	181
Curcumin	CSN5	Multiple cancers	Degradation	increases ubiquitination and decreases protein stability to downregulate PD-L1	182
ATRA+ATO	Pin1	Promyelocytic Leukemia	Degradation	decreases HIP1R-mediated lysosomal degradation of PD-L1	183
D-mannose	AMPK	Breast Cancer,Colorectal Cancer, Pancreatic Cancer, Bladder Cancer, Ovarian Cancer	Degradation	activates AMP-activated protein kinase (AMPK) to phosphorylate PD-L1 at S195, which leads to abnormal glycosylation and proteasomal degradation of PD-L1	46

AP1: activator protein 1; ATRA+ATO: all-trans retinoic acid plus arsenic trioxide; GSK3:glycogen synthase kinase 3; H3K4me3, histone H3 lysine 4 trimethylation; H3K27me3: histone H3 lysine 27 trimethylation; HNSCC: head and neck squamous cell carcinoma; NSCLC: non-small-cell lung cancer; dMMR/MSI-H: mismatch repair defect or High microsatellite instability; SHH: sonic hedgehog; STAT: signal transducer and activator of transcription; TAZ: WW domain-containing transcription regulator protein 1; TNBC: triple-negative breast cancer; YAP1, Yes-associated protein 1;SCLC:small cell lung cancer; UC: Urothelial carcinoma; HCC: hepatocellular carcinoma; RCC: renal cell carcinoma; ADCC: antibody-dependent cell-mediated cytotoxicity; FDA: Food and Drug Administration; NMPA: National Medical Products Administration; Refs: References.

## Abbreviations

ADC: antibody drug conjugate
ADCC: antibody-dependent cellular cytotoxicity
αFc: anti-IgG antibody
αFc-NP: a universal nanoparticle platform
AIEgen: aggregation-induced emission luminogen
ALP: alkaline phosphatase
AMPK: AMP-activated protein kinase
aPD-L1: anti-PD-L1 antibody
AuNDs: Gold nanodumbbells
BSA: bovine serum albumin
bsAbs: bispecific antibodies
Ce6: chlorine6
CIT: chemoimmunotherapy
cPD-L1: cytoplasmic PD-L1
CPP: cell-penetrating peptide
CTLA-4: cytotoxic T lymphocyte-associated antigen 4
DBCO: dibenzocyclooctyne
DCs: dendritic cells
DDR: DNA damage repair
DNAzyme: deoxyribozyme
DZ: DNAzyme
ERAD: endoplasmic reticulum associated degradation
EVs: extracellular vesicles
exoPD-L1: exosome surface PD-L1
EZH2: Enhancer of zeste 2
FAK: focal adhesion kinase
FDA: US Food and Drug Administration
HCC: hepatocellular carcinoma
HIP1R: Huntingtin interacting protein 1-related
H-MnO2: hollow-manganese dioxide
HPMA: N-(2-hydroxypropyl) methacrylamide
ICB: immune checkpoint blockade
ICD: immunogenic cell death
IPI549: PI3-Kinase γ inhibitor
irAEs: immune-related adverse effects
IRF1-de: IRF1-immunodepleted
LEXs: leukemia cell-derived exosomes
LNPs: lipid nanoparticles
mAb: monoclonal antibody
MHCs: major histocompatibility complexes
miRNA: MicroRNA
MM/GBSA: The molecular mechanics generalized Born surface area
mPD-L1: membrane PD-L1
MPNs: metal-phenolic networks
MPPA: multivalent HPMA polymer-peptide antagonists to PD-L1
MUC1-C: c-terminal subunit of mucin 1
NIR-II: the second near-infrared window
NMPA: National Medical Products Administration
Npd-L1: nuclear PD-L1
NR-IgG: normal rabbit IgG
NSCLC: non-small cell lung cancer
PD-1: programmed cell death 1
PD-L1: programmed cell death ligand 1
PDT: photodynamic therapy
PROTACs: proteolysis targeting chimeras
PTT: photothermal therapy
RCC: renal cell carcinoma
ROS: reactive oxygen species
sgRNA: single-strand guide RNA
shRNA: small hairpin RNA
siRNA: small interfering RNA
sMIC: the soluble NKG2D ligands
SMPAI: symbiotic mild photothermal-sensitized immunotherapy
sPD-L1: soluble PD-L1
TAAs: tumor-associated antigens
TGF-β: transforming growth factor-beta
TME: tumor microenvironment
VEGF: vascular endothelial growth factor
VP: verteporfin
